# ﻿A new Mexican species of the *Cryptopygus* complex (Collembola, Isotomidae) associated with the hermit crab *Coenobitaclypeatus* (Crustacea, Coenobitidae)

**DOI:** 10.3897/zookeys.1205.114394

**Published:** 2024-06-20

**Authors:** José G. Palacios-Vargas

**Affiliations:** 1 Laboratorio de Ecología y Sistemática de Microartrópodos, Departamento de Ecología y Recursos Naturales, Facultad de Ciencias, Universidad Nacional Autónoma de México, 04510 México, D.F., Mexico Universidad Nacional Autónoma de México México Mexico

**Keywords:** Sand, seashore, taxonomy, Xcacel

## Abstract

A new species of *Cryptopygus* Willem, 1901 associated with hermit crabs living on seashores of Quintana Roo State, Mexico, is described and illustrated. It is blind, with 9–11 postlabial setae, unguis with a pair of lateral teeth, empodial appendix lanceolate and almost as long as unguis, tenaculum with 4 + 4 teeth and 3–4 setae on corpus, manubrium with 11–14 pairs of manubrial setae on anterior surface and 17–18 pairs on posterior surface, and mucro bidentate. An updated key for the identification of 29 American species of *Cryptopygus* complex is included.

## ﻿Introduction

This contribution is a part of the project aimed at the microarthropods associated with hermit crabs, in which the male of *Coenaletescaribaeus* Bellinger, 1985 (Coenaletidae) has been redescribed (Palacios-Vargas et al. 2000) and other Collembola and some Acari recorded from this crab (Maldonado-Vargas and Palacios-Vargas 1999).

The genus *Cryptopygus* Willem, 1901 *sensu lato* represents a complex of genera (Potapov 2001; Potapov et al. 2013). The species of this complex live in a wide variety of environments such as soil, litter, caves, and sandy beaches. Here, I use the name *Cryptopygus* in its broad sense. There are altogether 76 valid species involved in the genus (Bellinger et al.1996–2023), which has a global distribution. The purpose of this paper is to describe a new species of *Cryptopygus* found in hermit crabs collected in marine waters of Quintana Roo State, Mexico. As shown by Palacios-Vargas (1997), there are several *Cryptopygus* species known from Mexico, namely *C.benhami* Christiansen & Bellinger, 1980 found in caves from Guerrero and Mexico States and *C.exilis* from Veracruz State. Later, Palacios-Vargas and Thibaud (2001) described an additional species, *Cryptopygusaxacayacatl* Vargas & Thibaud, 2001, which lives on sandy beaches from Guerrero State. Moreover, *Pauropyguscaussaneli* (Thibaud, 1996), which belongs to the same complex, was cited from Guerrero state by Potapov et al. (2013).

## ﻿Materials and methods

The material of the new species comes from Xcacel beach, Quintana Roo State. It was found while examining the hermit crab *Coenobitaclypeatus* (Fabricius, 1787) living in *Cittariumpica* (Linnaeus, 1758) shells. Hermit crabs were put in a bucket with fresh water and springtails floating on the surface were collected. The specimens were fixed in ethanol 96% and later cleared with KOH 10% and mounted on slides using Hoyer solution. To harden the solution, the slides were dried in a slide warmer at 45–50 °C for 1 week. Finally, each specimen was labeled with its collecting data. Specimens were examined with a Carl Zeiss Primo Star phase-contrast microscope. The drawings were made with the aid of a drawing tube.

Abbreviations:

**Abd** abdominal segment;

**Ant** antennal segment;

**PAO** postantennal organ;

**S** sensillum;

**Tita** tibiotarsus;

**Th** thoracic segment.

## ﻿Taxonomy

**Entomobryomorpha**

**Isotomidae**

**Anurophorinae**

### ﻿ Cryptopygus

Taxon classificationAnimaliaCollembolaIsotomidae

Willem, 1901

7248F52F-E622-5825-BFFD-F3D1CAE99F07

#### Diagnosis

**(modified from Potapov 2001).**Anurophorinae with fused Abd V and VI, well-developed furcula and bidentate or tridentate mucro. Slender body shape, from small to median size. Eyes from 0+0 to 8+8. Color usually pale. Integument smooth, with rare exceptions. Furcula present, mucro separated from dens, bi- or tridentate. Manubrium with 1–8 pairs of anterior setae. Dens slender, smooth or crenulated, continuously narrowing towards its apex. Abd V and VI fused. PAO and empodium present; anal spines absent, apical bulb usually absent on antenna. Clavate tibiotarsal hairs absent or present. Body setae usually short, macrosetae differentiated at least on last abdominal segments.

### ﻿ Cryptopygus coenobitus sp. nov.

Taxon classificationAnimaliaCollembolaIsotomidae

1CD4A58E-B5F8-5BA6-9C0E-A04B856BFD16

https://zoobank.org/41349B8A-6C87-41D9-B1E3-169668996F9A

Figs 1–4 , 5–8 , 9–12 , 13–16

#### Type material.

***Holotype*.** Female on slide. ***Paratype***: male on slide. Type material kept at Facultad de Ciencias, UNAM. Slides numbered: FC-UNAM:LESM-AC: 22863 and FC-UNAM:LESM-AC: 22864.

#### Type locality.

Mexico – Quintana Roo • Municipality of Solidaridad; Xcacel; ex. *Coenobitaclypeatus*; 20°20'13"N, 87°20'45"W; 6 June 2022; J.G. Palacios-Vargas, M. Ojeda & A. Arango leg.

#### Diagnosis.

White and eyeless *Cryptopygus*, with 9+9 to 11+11 postlabial setae, 1 pair of lateral teeth on unguis; retinaculum with 4 + 4 teeth and 3–4 setae on corpus, 11–14 pairs of manubrial setae on anterior surface, and 17–18 pairs on posterior surface, dens smooth, 4 setae on posterior basal part and 11–13 on anterior side, mucro bidentate.

#### Description.

**Figures 1–4.**
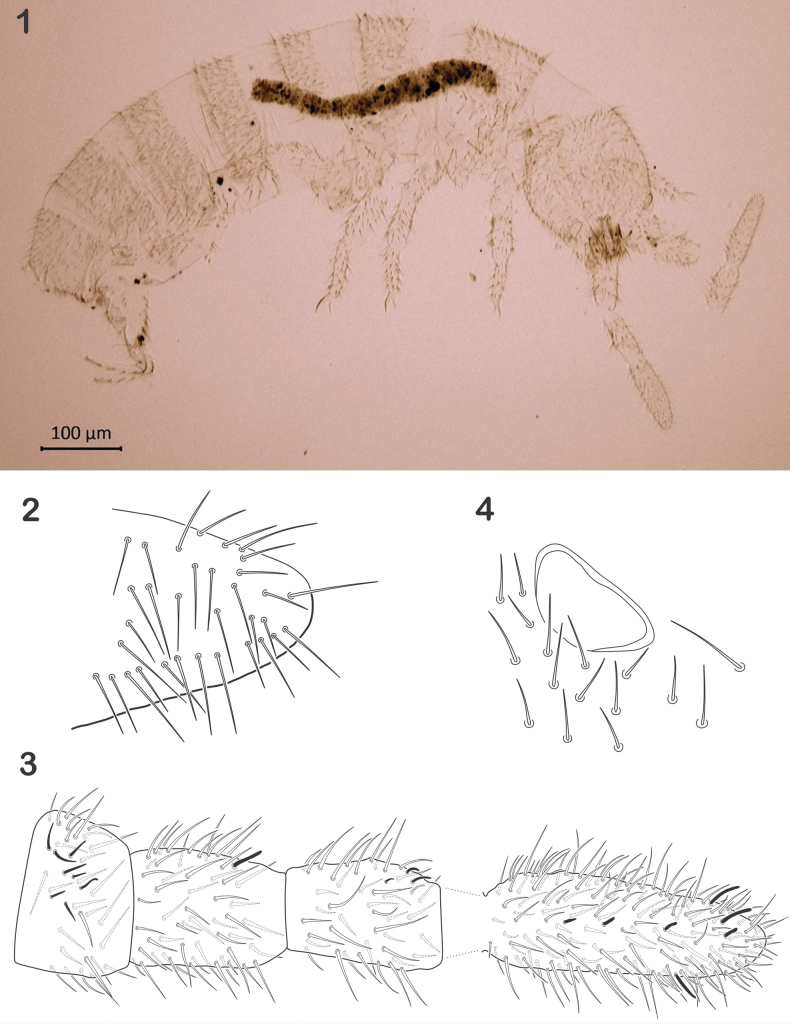
*Cryptopyguscoenobitus* sp. nov. **1** habitus, image from phase-contrast microscope, lateral view **2**Abd II, lateral partial view **3** antenna I–IV, from I to III in ventral view, IV dorsal view **4**PAO.

Setae smooth, acuminate, mostly short, some micro- and few slightly barbulated macrosetae (40–50 µm). Sensilla on terga in posterior row of setae, only slightly differentiated from ordinary setae, thinner and hyaline (Figs 1, 2). Microsensillum on Th. II normal. Abd. V and VI completely fused. Dorsal setation from Th I to Abd IV, 15,8/4,4,5,8 irregular lines (Fig. 1). Thorax without ventral setae. Abd VI without foil setae. Sensillar formula from Th II to Abd V: 3,3/2,2,2,3,2.

**Figures 5–8.**
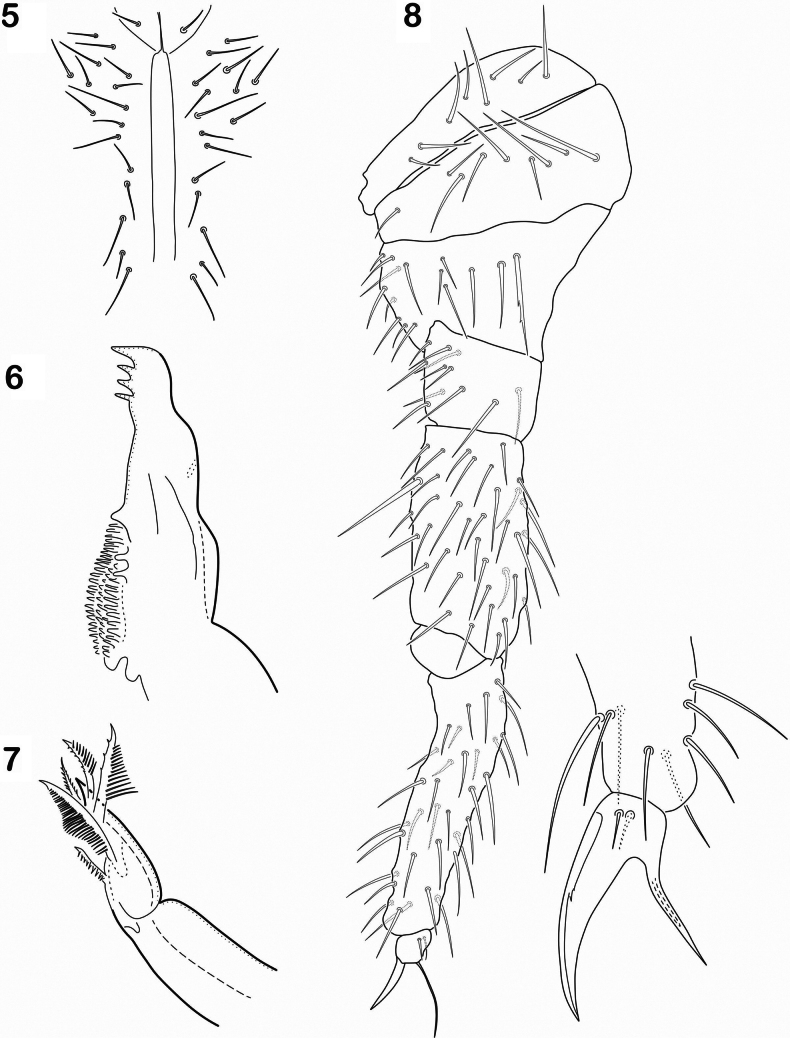
*Cryptopyguscoenobitus* sp. nov. **5** postlabial setae **6** mandible **7** maxilla **8** tita III and magnification of foot complex.

Length of head 140–150 µm, length of body 750–1000 µm. Antennae 250 µm, longer than head (ratio = 1:1.75). Ratio Ant. I–IV as 1:1.5; 1.5; 2.5. Ant. I with 33–36 setae, 9 thin and short ventral sensilla; Ant. II with 54 setae with few microsensilla and one thick, long sensillum on ventral–distal position (Fig. 3). Ant. III with 44 setae, antennal organ III with 2 microsensilla, 2 fine guard sensilla and 1 ventro-external microsensillum. Ant IV with about 70 setae, and 8 long and fine sensilla. Apex of the segment with a microsensillum and a small organite, apical bulb absent. Eyes absent, postantennal organ oval (25 × 12 µm), surrounded by 5 setae (Fig. 4). Labrum with 4 short, thin prelabral setae; 4 long setae in anterior row, 5 spiniform setae in median row, and 4 spiniform setae in posterior row, formula 4/4,5,4. With 4 sublobal hairs on outer maxillary lobe; labium with full set of guards on papillae A–E, and 3 proximal and 4 baso-median setae. Postlabial setae 9+9-11+11 (Fig. 5). Mandibles with 3–4 apical teeth and molar surface well developed (Fig. 6). Maxilla with 6 lamellae, with some plumose (Fig. 7).

**Figures 9–12.**
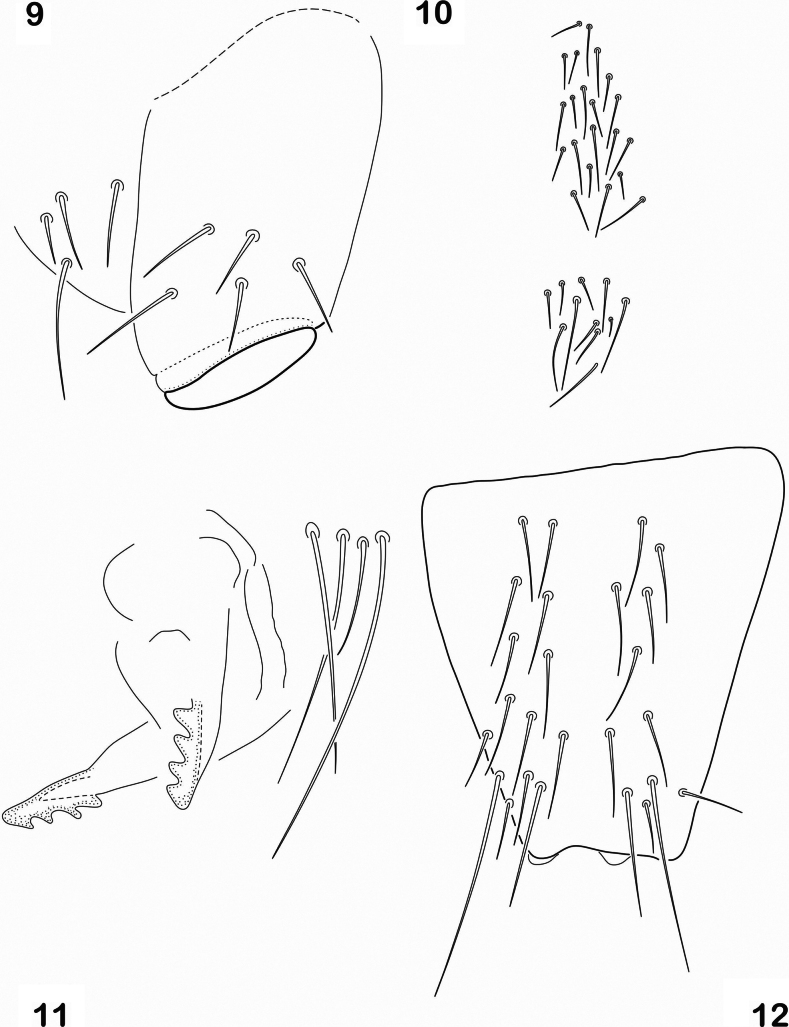
*Cryptopyguscoenobitus* sp. nov. **9** ventral tube, lateral view **10** coxae furcalis anterior and posterior **11** tenaculum **12** manubrium, anterior view.

Thorax without ventral setae. All femora with 1 ventral acuminate tenent hair. Tita with 2,3,3 acuminate tenent hairs and without a distal subsegment (Fig. 8). Tita I = 60 µm, Tita = 80 µm. Unguis I = 13 µm, unguiculus I = 11 µm. Unguis with 1 pair of lateral teeth in the median part. Unguiculus lanceolate, acuminate, not lamellate, almost as long as unguis. Ventral tube with 4 + 4 basal setae and 5 + 5 latero-distal setae (Fig. 9). Abd. IV on ventral surface with 4+5 setae by side; anterior subcoxa furcalis with 19–20 setae; posterior subcoxa furcalis with 10 setae, 4 of them longer than others (Fig. 10).

**Figures 13–16.**
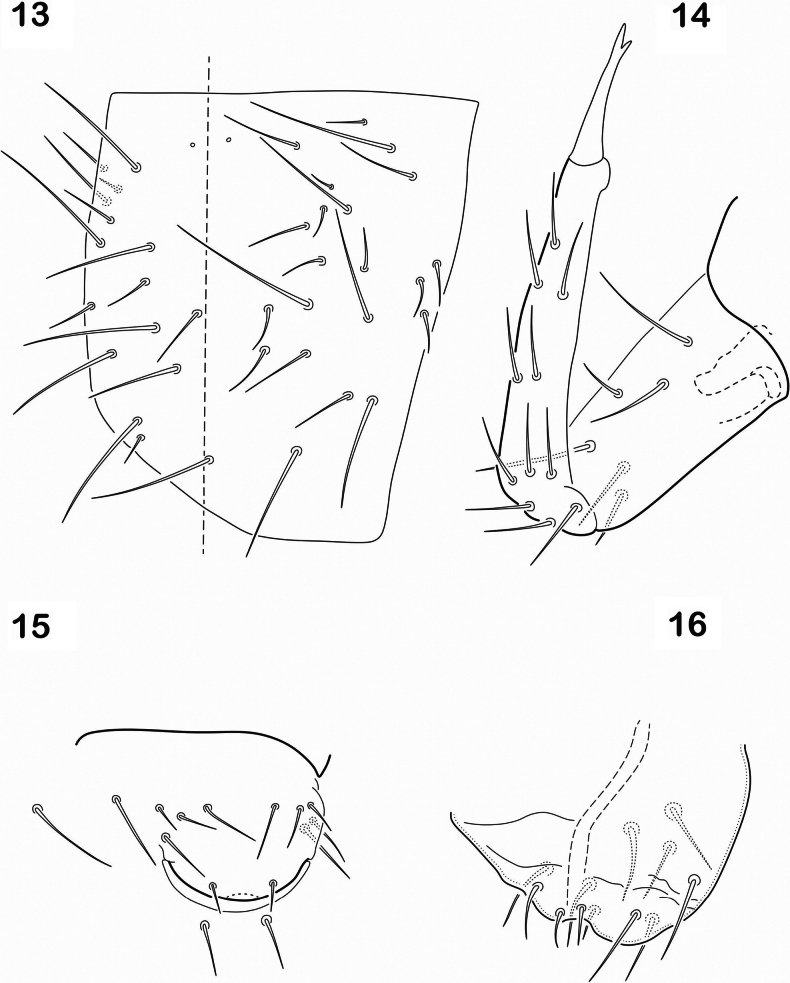
*Cryptopyguscoenobitus* sp. nov. **13** manubrium latero-posterior view, line shows midline **14** dens and mucro **15** female genital plate **16** male genital plate.

Tenaculum with 4+4 teeth, corpus with 3–4 setae (Fig. 11). Furcula well developed, manubrium with 11–14 pairs of anterior setae (Fig. 12), 3 small lateral setae and 18 pairs of setae on posterior side. (Fig. 13). Dens smooth, only 4 setae of different size on posterior basal part and 10–14 subequal setae on anterior surface (Fig. 14). Manubrium 80 µm, dens 100 µm, mucro 25 µm. Ratio 1:1.25; 0.3. Mucro bidentate. Genital plate of female with 2 eugenital setae and 5 pairs of pregenital setae (Fig. 15). Male with 4 pairs of eugenital setae and a few circumgenital setae (Fig. 16).

#### Etymology.

This species is a noun in apposition after the genus of the hermit crab in which it was found.

#### Distribution.

*Cryptopyguscoenobitus* sp. nov. is only known from Xcacel, Quintana Roo State, Mexico.

### ﻿Key to *Cryptopygus* complex s. l. from the Americas

(some genera are excluded; they are not common, or descriptions are too brief)

**Table:** 

1	Mucro quinquedentate (3 teeth and 2 basales spines), blind and colorless, body length 0.7 mm	***Mucrosomiacaeca* Wahlgren, 1906** (South Georgia, Argentina, Chile, Peru, Venezuela; general distribution: widely in Antarctic and Subantarctic)
–	Mucro bi- or tridentate, eyes and color present or absent	**2**
2	Foil setae on the end of fused tergite V+VI present	**3 (*Hemisotoma*)**
–	Foil setae on the end of fused tergite V+VI absent	**4**
3	Body length 1.0–1.2 mm, 8–7 eyes per side, ungual tooth present	***H.thermophila* (Axelson, 1900)** (cosmopolitan)
–	Body length 0.9 mm, 5 eyes per side, ungual tooth absent	***H.similis* (James, 1933)** (Canada)
4	Mandible reduced and elongated, molar plate with 2 strong basal teeth, antenna set together on frontal part of head, PAO large and wide	***Pauropyguscaussaneli* (Thibaud, 1996)** (Mauritania, Mexico Pacific cost; sandy seashores of the tropics and subtropics)
–	Mandible normal, not elongated, molar plate without strong basal teeth, antenna set separate on head, PAO elongated and narrow	**5**
5	Conspicuous flattened sensilla on fused abdominal tergite V+VI	**6 (*Proisotomodes*)**
–	Flattened sensilla on fused abdominal tergite V+VI absent	**7**
6	Eyes absent, Ant I with 1 thick sensillum	***P.axayacatl* Palacios-Vargas & Thibaud, 2001** (Mexico Pacific coast)
–	1+1 eyes, Ant II with 2 thick sensilla	***P.bipunctatus* (Axelson, 1903)** (USA, Europe; general distribution: Holarctic)
7	Eyes and pigment present	**11**
–	Eyes and pigment absent	**8**
8	Manubrium with 11–14 pairs of anterior setae, unguis with 2 lateral teeth	***Cryptopyguscoenobitus* sp. nov.** (Mexico)
–	Manubrium with 1–2 pairs of anterior setae, unguis without lateral teeth	**9**
9	Unguis with 1 tooth	***C.benhami* Christiansen & Belinger, 1980** (USA, Mexico)
–	Unguis without any tooth	**10**
10	Manubrium with 2 anterior and 8 posterior pairs of setae	***C.elegans* (Rapoport & Izarra, 1962)** (Argentina)
–	Manubrium with 1 anterior pair of setae	**13**
11	8+8 eyes	**20**
–	6+6 or fewer eyes	**12**
12	2+2 eyes	**13**
–	3+3 to 6+6 eyes	**15**
13	Body length about 2 mm, ventral tube with 7 basal and 8+8 distal setae	***C.arcticus* Christiansen & Bellinger, 1980**
–	Body length 1 mm, ventral tube with 1 basal seta and 4+4 distal setae	**14**
14	Mucro with 3 teeth	***C.parallelus* (Wahlgren, 1901)** (Chile)
–	Mucro with 2 teeth	***C.quadrioculatus* (Rapoport, 1963)** (Argentina)
15	3+3 eyes per side	**16**
–	With more eyes per side	**17**
16	Body 1.7 mm, PAO very long, 5–6 times longer than 1 eye `	***C.insignis* Massoud & Rapoport, 1968** (Chile, Argentina)
–	Body 0.6 mm, PAO short, 3 times longer than 1 eye	***C.trioculatus* Izarra, 1972** (Argentina)
17	5+5 eyes per side	**18**
–	6+6 eyes per side	**24**
18	Mucro with 3 teeth, unguis without teeth, length up to 1.25 mm	***C.patagonicus* Izarra, 1972** (Argentina)
–	Mucro with 2 teeth, unguis with or without teeth, length less than 1 mm	**19**
19	Dens with many anterior and posterior setae	***C.decemoculatus* Folsom, 1937** (USA)
–	Dens with only 5 anterior and 4 posterior setae	***C.quinqueoculatus* Izarra, 1970** (Argentina)
20	Body length less than 1 mm, Ant III with 9 sensilla	***C.indecisus* Massoud & Rapoport, 1968** (Argentina, Chile)
–	Body length more than 1.4 mm, Ant III with only 5 sensilla	**21**
21	Unguis without teeth	**22**
–	Unguis with teeth	**23**
22	Manubrium with 2–3 pairs of ventral setae; dens with 4 anterior and 9 posterior setae	***C.ambus* Christiansen & Bellinger, 1980** (USA)
–	Manubrium with 1 pair of ventral setae; dens with 0 anterior and 18 posterior pairs of setae	***C.pentatomus* (Börner, 1906)** (Brazil)
23	Unguis with 1 median tooth	***C.aquae* (Bacon, 1914)** (USA, Canada, Pacific coast)
–	Unguis with 1 median and 2 lateral teeth	***C.araucanus* Massoud & Rapoport, 1968** (Argentina, Chile)
24	Body length 2 mm	**25**
–	Body length less than 2 mm	**26**
25	Unguis with 2 lateral teeth	***C.antarcticus* Willem, 1901** (Antarctic, Argentina)
–	Unguis without lateral teeth	***C.cinctus* Wahlgren, 1906** (Argentina, Chile)
26	Body length about 1.5 mm	**27**
–	Body length less than 1 mm	**28**
27	Ventral tube with 2 basal and 4+4 distal setae; dens with 20 anterior and 19 posterior setae	***C.separatus* Denis, 1931** (Costa Rica)
–	Ventral tube with 6 basal and 4+4 distal setae; dens with 5 anterior and 6 posterior setae	***C.ulrikeae* (Najt & Thibaud, 1987)** (Ecuador)
28	Dens with 6 anterior and 13 pairs of setae	***C.andinus* Díaz & Najt, 1995** (Venezuela)
–	Dens with 6 anterior and 4 pairs of setae	***C.hirsutus* (Denis, 1931)** (Costa Rica, Peru)
29	Dens with 7 anterior and 2 posterior setae, mucro with 2 teeth	***C.exilis* (Gisin, 1960)** (USA, Mexico, Europe)
–	Manubrium with 4 pairs of anterior and 3 posterior setae, mucro with 3 teeth	***Cryptopygusyosiii* Izarra, 1965** (Argentina)

## ﻿Discussion

*Cryptopyguscoenobitus* sp. nov. is similar to *Proisotomodesaxayacatl*, *Mucrosomiacaeca*, *Cryptopygusbenhami*, *C.elegans*, *C.exilis*, *C.yosiii*, and *Pauropyguscaussaneli* in lacking eyes and pigment, but these species have four basal setae and 4+4 setae on ventral tube (vs 8 basal and 5+5 in the new species) and four teeth on the ramus of the tenaculum and one seta on the corpus (vs 3–4 on corpus in the new species). Most species have one or two pairs of setae on the anterior side of the manubrium, in contrast to 18 pairs in the new species. Moreover, the new species has many postlabial setae (9–11 pairs), an unguis with a pair of lateral teeth but without an internal tooth, a lanceolate empodium that is almost as long as the unguis, a manubrium with 8+8 anterior setae, and a tridentate mucro with two basal spines.

When *C.coenobitus* sp. nov. is compared with *Isotominellalaterochaeta* from South Africa, there are some characters common, such as the lack of eyes and pigment and the presence of numerous setae on ventral side of head, mainly along the ventral line. The furcula is also very similar in shape, there are four setae on posterior basal part and 11–13 on anterior surface, and the mucro is bidentate. The main differences are that *C.coenobitus* lacks ventral setae on thorax III, and the ventral tube has 5 + 5 laterodistal and 4 + 4 posterior setae (vs 5–8+5–8 laterodistal and 6–9 posterior in *I.laterochaeta*). The tenaculum has 4+4 teeth, and the corpus bears three or four setae (vs 4+4 teeth and 2 setae on corpus).

There are some important characters that have been omitted in the descriptions of many species of the *Cryptopygus* complex, such as (1) postlabial chaetotaxy, which usually represents four or five pairs of setae, (2) the shape of maxillary lamellae, which can be modified depending on food preferences, and (3) the setation of coxae furcalis, but this last charactive might have important diagnostic value.

*Cryptopyguscoenobitus* sp. nov. seems to be psammobiotic, and its morphology does not appear modified for living in association with hermit crabs. The maxillae are plumose, as an adaptation for filtration of food particles from the water, and the mandibles are normal, not modified, as in *Coenaletes* spp. (Coenaletidae) which live between the *Cittariumpica* shell and the hermit crab occupant. The new *Cryptopygus* species has been found in the sand but is able to enter to the shells occupied by hermit crabs, as in other Collembola genera such as *Cyphoderus* and additional microarthropods as found by Maldonado-Vargas and Palacios-Vargas (1999).

## Supplementary Material

XML Treatment for Cryptopygus

XML Treatment for Cryptopygus coenobitus

## ﻿Additional information

### Author ORCIDs

José G. Palacios-Vargas https://orcid.org/0000-0001-9097-6813

### Data availability

All of the data that support the findings of this study are available in the main text.
